# Low body temperature and mortality in critically ill patients with coronary heart disease: a retrospective analysis from MIMIC-IV database

**DOI:** 10.1186/s40001-023-01584-8

**Published:** 2023-12-20

**Authors:** Weiran Luo, Lixue Cao, Chuan Wang

**Affiliations:** 1 The Six Clinical Medical School, Capital Medical University, Beijing, China; 2 Department of Medical Genetics and Developmental Biology, Capital Medical University School of Basic Medical Sciences, Beijing, China; 3 Department of Cardiac Surgery, Beijing Anzhen Hospital, Capital Medical University, 2 Anzhen Road, Beijing, 100029 China

**Keywords:** Body temperature, Intensive care unit, Clinical outcome, Coronary heart disease

## Abstract

**Background:**

This study was aimed to investigate the correlation between low body temperature and outcomes in critically ill patients with coronary heart disease (CHD).

**Methods:**

Participants from the Medical Information Mart for Intensive Care (MIMIC)-IV were divided into three groups (≤ 36.5 ℃, 36.6–37.4 ℃, ≥ 37.5 ℃) in accordance with body temperature measured orally in ICU. In-hospital, 28-day and 90-day mortality were the major outcomes. Multivariable Cox regression, decision curve analysis (DCA), restricted cubic splines (RCS), Kaplan–Meier curves (with or without propensity score matching), and subgroup analyses were used to investigate the association between body temperature and outcomes.

**Results:**

A total of 8577 patients (65% men) were included. The in-hospital, 28-day, 90-day, and 1-year overall mortality rate were 10.9%, 16.7%, 21.5%, and 30.4%, respectively. Multivariable Cox proportional hazards regression analyses indicated that patients with hypothermia compared to the patients with normothermia were at higher risk of in-hospital [adjusted hazard ratios (HR) 1.23, 95% confidence interval (CI) 1.01–1.49], 28-day (1.38, 1.19–1.61), and 90-day (1.36, 1.19–1.56) overall mortality. For every 1 ℃ decrease in body temperature, adjusted survival rates were likely to eliminate 14.6% during the 1-year follow-up. The DCA suggested the applicability of the model 3 in clinical practice and the RCS revealed a consistent higher mortality in hypothermia group.

**Conclusions:**

Low body temperature was associated with increased mortality in critically ill patients with coronary heart disease.

**Supplementary Information:**

The online version contains supplementary material available at 10.1186/s40001-023-01584-8.

## Background

Even though significant advancements in care have rendered declined life lost, coronary heart disease (CHD) continues to be a leading cause of death and premature death, resulting in great healthcare expenditure [1]. Critically ill patients in the intensive care unit (ICU) often have complex underlying causes, with cardiac surgery for CHD being the primary reason for ICU admission in over 45% of cases. Furthermore, the mortality rate for patients experiencing exacerbation of chronic cardiovascular disease within 1 year after admission was found to be 16.1%, making it the second highest cause of death following malignant tumors [2]. Therefore, the role of predictive factors, biomarkers, and scores in the development and prognosis of CHD has been widely acknowledged [3–7]. Although these factors are of good efficiency in predicting, some of them are too complicated to be routinely used and postponed awaiting laboratory tests.

Body temperature, a crucial physiological parameter frequently measured in ICU, affects inflammation and immune function, related to various diagnoses caused by infectious or not [8, 9]. In the operating room setting, rectal, bladder, esophageal, and nasopharyngeal probes are preferred for monitoring core temperature, while the invasive measurement of temperature in the pulmonary artery, considered the gold standard, is rarely employed. The skin temperature detected by infrared thermometers, which is highly susceptible to changes in ambient air temperature, thereby is not commonly used in ICU [10]. Compared with that, alternative peripheral temperature measurements, such as oral temperature, demonstrate comparable precision to the nasopharyngeal one (*P* = 1.00) with better acceptance in ICU [11]. An evidence-based guideline [12] also suggested that the study involving patients with CHD measuring peripheral temperature to predict morbid cardiac events by multivariate analysis was of good quality. In addition to that, another guideline [13] from The American Society of PeriAnesthesia Nurses (ASPAN) showed strong evidence for oral temperature measurements, with a recommendation class of Class I, Level B.

Whether temperature abnormalities have an influence on CHD patients remains unknown yet, so we hypothesized low body temperature was linked with worse outcome based on similar investigations aiming at patients undergoing coronary artery bypass grafting (CABG) [14, 15] and other cardiac surgeries [16]. And we tested this in the Medical Information Mart for Intensive Care (MIMIC)-IV database in a pre-specified manner.

## Methods

### Study population

The Medical Information Mart for Intensive Care (MIMIC)-IV database (version 2.2) was the data source of the present retrospective observational study, containing 73,181 ICU admission records from critically ill patients in the Beth Israel Deaconess Medical Center (BIDMC) from 2008 to 2019 [17–19]. The access to the database was obtained based on both the training named ‘CITI Data or Specimens Only Research’ passed (record ID: 57,385,572) and the application for credentialed access of PhysioNet Clinical Databases approved. Moreover, the waiver of informed consent was granted by the Institutional Review Board at the Beth Israel Deaconess Medical Center and the data in the database were de-identified.

We enrolled 8577 ICU patients diagnosed with coronary heart disease (CHD) based on the International Classification of Diseases (ICD)-10 codes from I20 to I25. Patients with censored body temperature records had been excluded, and the overall study population was divided into three groups in accordance with the prior investigation [20] and the distribution of body temperature a priori.

### Definitions of body temperatures and outcomes

The body temperature was defined as an average body temperature (derived from sources of oral thermometer) within 24 h after ICU admission and the follow-up of mortality kicked off on the date of discharge.

### Data acquisition

PostgreSQL 15.4 (PostgreSQL Global Development Group, Berkley, California, USA) was employed to collect data through structure query language (SQL) and contents were extracted as follows: demographic data (age, gender and ethnicity), patient history (atrial fibrillation, chronic kidney disease, chronic obstructive pulmonary disease or pulmonary hypertension, diabetes mellitus, hyperlipidemia, hypertension, prior myocardial infarction, previous cardiac surgery, heart arrest and cardiogenic shock), laboratory results (white blood cell, hemoglobin, hematocrit, platelet, serum creatinine, glucose, blood urea nitrogen, international normalized ratio (INR), lactate, potassium and sodium), admission type, first care unit [Coronary Care Unit (CCU), Cardiac Vascular Intensive Care Unit (CVICU), Medical Intensive Care Unit (MICU), Medical/Surgical Intensive Care Unit (MICU/SICU), Neuro Intermediate, Neuro Stepdown, Neuro Surgical Intensive Care Unit (Neuro SICU), Surgical Intensive Care Unit (SICU), Trauma SICU (TSICU)], vital signs (systolic blood pressure, diastolic blood pressure, mean blood pressure, heart rate and respiratory rate), vasoactive drugs used during ICU stay (dobutamine, dopamine, epinephrine, milrinone, norepinephrine, vasopressin and phenylephrine) and types of CHD [21] (acute coronary syndrome and stable CHD). Score system (SOFA and SAPS II) was obtained in the aid of codes in MIMIC Code Repository (https://github.com/MIT-LCP/mimic-code). Missing data were less than 1.5% (Additional File 1: Table S1).

### Statistical analysis

Categorical variables are compared using the Chi-Square test, illustrated in the form of number and percentage and continuous variables using one-way analysis of variance (ANOVA) reported as mean ± standard deviation. To evaluate the independent association of body temperature and in-hospital, 28-day and 90-day mortality, multiple models were under adjustment of confounding factors which had been analyzed in univariate analysis models with *P* < 0.05: Model 1: adjusted for age, gender, ethnicity; Model 2: adjusted for model 1 plus types of coronary heart disease, admission type, white blood cell, hemoglobin, hematocrit, serum creatinine, glucose, blood urea nitrogen, INR, potassium, systolic blood pressure, diastolic blood pressure, mean blood pressure, heart rate, respiratory rate, SOFA and SAPS II; Model 3: adjusted for model 2 plus atrial fibrillation, chronic kidney disease, chronic obstructive pulmonary disease or pulmonary hypertension, heart arrest, cardiogenic shock and vasoactive drugs (dobutamine, dopamine, epinephrine, milrinone, norepinephrine, vasopressin and phenylephrine). Decision curve analysis (DCA) was performed to evaluate the predictive effect between three multiple models. Kaplan–Meier survival curves were compared among three body temperature groups using the log-tank test and the body temperature was also regarded as a continuous variable to scope a probable non-linear relationship with mortality using restricted cubic splines (RCS). Subgroup analysis was performed in terms of age, gender, ethnicity, heart arrest and cardiogenic shock to figure out if the interactions between body temperature as a continuous variable and these variables function.

## Results

After screening of critically ill patients diagnosed as CHD with complete records of body temperature during ICU stay and other admission data, 8577 patients were eventually enrolled in the present analysis cohort with the mean age of 69 years. Of these, 65% were men. Enrolled patients were distributed into three groups (℃): hypothermia (31.8–36.5), normothermia (36.6–37.4), and hyperthermia (37.5–39.6). The distribution of temperature was illustrated (Additional File 1: Figure S1). The in-hospital, 28-day, 90-day, 180-day and 1-year overall mortality rate were 10.9%, 16.7%, 21.5% and 30.4%, respectively.

### Baseline characteristics

Baseline characteristics of the study population are showcased in Table 1. Compared with the other two groups, patients with hypothermia presented to own a higher proportion of older individuals, white, with atrial fibrillation, chronic kidney disease, cardiogenic shock, acute coronary syndrome (ACS, definition comes from ICD-10 codes [22]) and several vasoactive drugs (dobutamine, dopamine, milrinone and phenylephrine). The mortality of this group kept its leading role during 1-year follow-up with a steady increase in comparison with normothermia group.

**Table 1:** Baseline characteristics according to different groups of body temperature among critically ill patients with coronary heart disease

Variables	Hypothermia (N = 1064)	Normothermia (N = 7076)	Hyperthermia (N = 437)	*P* value
Patient characteristics				
Age, years (SD)	72.78 (11.78)	68.46 (12.68)	64.79 (13.20)	< 0.001
Male, n (%)	683 (64.2)	4657 (65.8)	273 (62.5)	0.238
White, n (%)	774 (72.7)	4862 (68.7)	256 (58.6)	< 0.001
Patient history, n (%)				
Atrial fibrillation	578 (54.3)	3135 (44.3)	188 (43.0)	< 0.001
Chronic kidney disease	503 (47.3)	2881 (40.7)	161 (36.8)	< 0.001
COPD or PH	352 (33.1)	2418 (34.2)	126 (28.8)	0.064
Cardiogenic shock	183 (17.2)	719 (10.2)	66 (15.1)	< 0.001
Diabetes mellitus	509 (47.8)	3576 (50.5)	224 (51.3)	0.236
Hyperlipidemia	271 (25.5)	1778 (25.1)	108 (24.7)	0.949
Hypertension	269 (25.3)	1766 (25.0)	109 (24.9)	0.974
Heart arrest	85 (8.0)	555 (7.8)	49 (11.2)	0.042
Prior myocardial infarction	412 (38.7)	2688 (38.0)	160 (36.6)	0.744
Previous cardiac surgery	312 (29.3)	1810 (25.6)	103 (23.6)	0.017
Laboratory tests				
BUN, mg/dL (SD)	35.23 (29.09)	29.69 (22.51)	28.72 (19.31)	< 0.001
Glucose, mg/dL (SD)	140.54 (57.41)	143.25 (55.86)	162.75 (74.43)	< 0.001
Hematocrit, % (SD)	31.34 (5.51)	31.41 (5.68)	31.94 (5.99)	0.181
Hemoglobin, g/dL (SD)	10.13 (1.85)	10.16 (1.95)	10.27 (2.01)	0.437
INR (SD)	1.59 (0.87)	1.46 (0.72)	1.53 (0.75)	< 0.001
Lactate, mg/dL (SD)	2.47 (1.85)	2.11 (1.37)	2.37 (1.73)	< 0.001
Potassium, mEq/L (SD)	4.39 (0.61)	4.34 (0.55)	4.27 (0.60)	0.002
Sodium, mEq/L (SD)	137.61 (4.89)	138.18 (4.50)	139.23 (5.30)	< 0.001
Serum creatinine, mg/dL (SD)	1.75 (1.67)	1.64 (1.63)	1.72 (1.50)	0.102
Platelet, K/µL (SD)	180.21 (87.47)	192.26 (88.64)	189.51 (101.12)	< 0.001
WBC, K/µL (SD)	11.95 (5.88)	12.57 (8.43)	14.05 (9.00)	< 0.001
ICU type, *n* (%)				< 0.001
Coronary Care Unit (CCU)	239 (22.5)	1193 (16.9)	54 (12.4)	
Cardiac Vascular Intensive Care Unit (CVICU)	403 (37.9)	2144 (30.3)	53 (12.1)	
Medical Intensive Care Unit (MICU)	140 (13.2)	1219 (17.2)	115 (26.3)	
Medical/Surgical Intensive Care Unit (MICU/SICU)	125 (11.7)	882 (12.5)	69 (15.8)	
Neuro Intermediate	19 (1.8)	227 (3.2)	5 (1.1)	
Neuro Stepdown	10 (0.9)	116 (1.6)	3 (0.7)	
Neuro Surgical Intensive Care Unit (Neuro SICU)	24 (2.3)	253 (3.6)	48 (11.0)	
Surgical Intensive Care Unit (SICU)	59 (5.5)	561 (7.9)	43 (9.8)	
Trauma SICU (TSICU)	45 (4.2)	481 (6.8)	47 (10.8)	
Admission type, n (%)				< 0.001
Elective	37 (3.5)	210 (3.0)	3 (0.7)	
Emergency	333 (31.3)	2379 (33.6)	199 (45.5)	
Urgent	287 (27.0)	1555 (22.0)	106 (24.3)	
Other	407 (38.3)	2932 (41.4)	129 (29.5)	
Vital sign, n (%)				
SBP	113.53 (14.45)	117.82 (15.70)	116.54 (15.92)	< 0.001
DBP	60.51 (10.55)	62.36 (10.55)	62.27 (10.76)	< 0.001
MBP	76.04 (9.73)	78.29 (10.34)	77.81 (10.63)	< 0.001
Heart rate	79.93 (14.50)	82.32 (14.32)	91.45 (16.34)	< 0.001
Respiratory rate	18.96 (3.33)	19.29 (3.32)	21.39 (3.74)	< 0.001
Type of CHD, n (%)				< 0.001
Acute coronary syndrome	963 (90.5)	6332 (89.5)	331 (75.7)	
Stable coronary heart disease	101 (9.5)	744 (10.5)	106 (24.3)	
Vasoactive drugs during ICU stay, n (%)				
Dobutamine	81 (7.6)	179 (2.5)	22 (5.0)	< 0.001
Dopamine	46 (4.3)	194 (2.7)	17 (3.9)	0.010
Epinephrine	99 (9.3)	460 (6.5)	51 (11.7)	< 0.001
Milrinone	38 (3.6)	109 (1.5)	4 (0.9)	< 0.001
Norepinephrine	279 (26.2)	1717 (24.3)	219 (50.1)	< 0.001
Phenylephrine	282 (26.5)	1584 (22.4)	104 (23.8)	0.011
Vasopressin	112 (10.5)	568 (8.0)	98 (22.4)	< 0.001
Score system (SD)				
SOFA	5.83 (3.61)	5.28 (3.46)	7.60 (3.96)	< 0.001
SAPS II	40.89 (12.75)	37.81 (12.45)	44.27 (15.10)	< 0.001
Outcome, n (%)				
In-hospital mortality	152 (14.3)	677 (9.6)	106 (24.3)	< 0.001
28-day mortality	245 (23.0)	1054 (14.9)	132 (30.2)	< 0.001
90-day mortality	312 (29.3)	1385 (19.6)	148 (33.9)	< 0.001
1-year mortality	409 (38.4)	2014 (28.5)	181 (41.4)	< 0.001

COPD, chronic obstructive pulmonary disease; PH, pulmonary hypertension; WBC, white blood cell; BUN, blood urea nitrogen; INR, international normalized ratio; SBP, systolic blood pressure; DBP, diastolic blood pressure; MBP, mean blood pressure; SOFA, sequential organ failure assessment; SAPS, simplified acute physiology score; SD, standard deviation

### Association of body temperature with clinical outcomes and evaluation of predictive model

The Cox proportional hazards regression model was adopted to analyze the association between low body temperature and clinical outcomes among critically ill patients with CHD (Table 2). On the whole, patients with hypothermia from in-hospital mortality to 90-day mortality were at a statistically higher risk than the normothermia group no matter for any multivariable model. Full-variable model 3 also indicated that there is no statistically difference between hyperthermia and normothermia.

**Table 2:** Cox proportional hazard ratios (HR) for outcomes of critically ill patients with coronary heart disease

Outcomes	Model 1			Model 2			Model 3		
	HR	95% CI	*P*-value	HR	95% CI	*P*-value	HR	95% CI	*P*-value
In-hospital mortality									
Hypothermia	1.37	1.14–1.63	< 0.001	1.23	1.02–1.49	0.033	1.23	1.01–1.49	0.040
Normothermia	1.00 (ref)	–	/	1.00 (ref)	–	/	1.00 (ref)	–	/
Hyperthermia	2.95	2.40–3.63	< 0.001	1.27	1.01–1.59	0.042	1.25	0.99–1.57	0.060
28-day mortality									
Hypothermia	1.40	1.22–1.61	< 0.001	1.38	1.18–1.60	< 0.001	1.38	1.19–1.61	< 0.001
Normothermia	1.00 (ref)	–	/	1.00 (ref)	–	/	1.00 (ref)	–	/
Hyperthermia	2.54	2.12–3.05	< 0.001	1.18	0.96–1.44	0.112	1.16	0.95–1.42	0.144
90-day mortality									
Hypothermia	1.38	1.22–1.56	< 0.001	1.35	1.18–1.55	< 0.001	1.36	1.19–1.56	< 0.001
Normothermia	1.00 (ref)	–	/	1.00 (ref)	–	/	1.00 (ref)	–	/
Hyperthermia	2.22	1.88–2.64	< 0.001	1.11	0.92–1.34	0.273	1.10	0.91–1.33	0.336

**Fig. 1:**
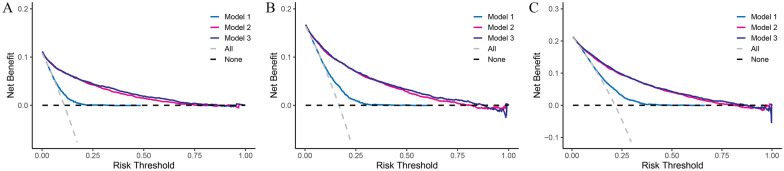
The decision curve analysis (DCA) to evaluate the predictive power of multiple models. **A** The DCA for in-hospital mortality; **B** the DCA for 28-day mortality; **C** the DCA for 90-day mortality

The full model (model 3) demonstrated a higher net benefit than model 1 and model 2 in assessing the prognosis of CHD patients as the risk threshold ranging from less than 0.05 to more than 0.75 for in-hospital, 28-day and 90-day mortality (Fig. 1). This was clinically adoptable due to its permission for a wide range of critically ill population with mortality rates ranging from a large scale.

**Fig. 2:**
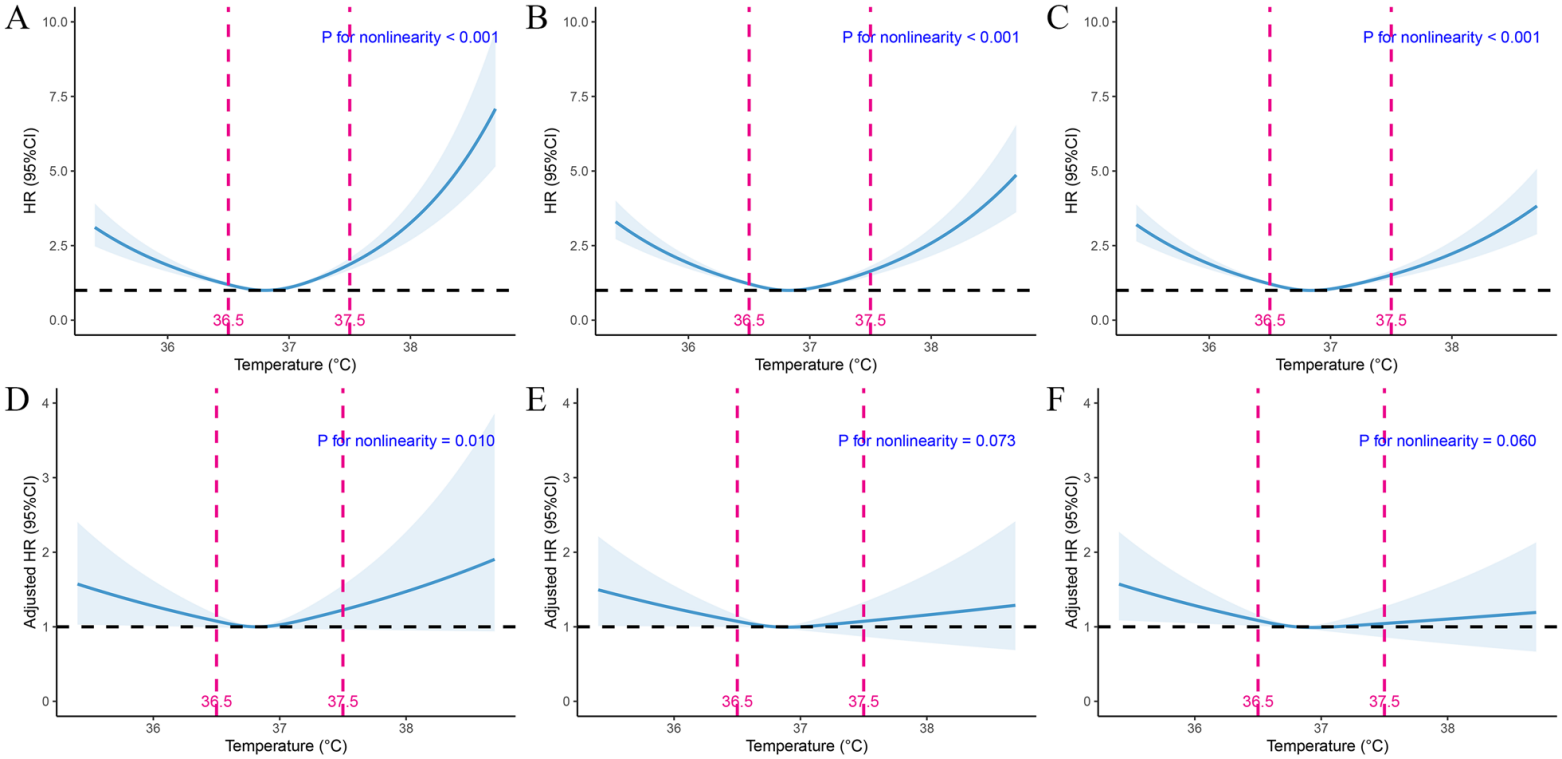
Association between body temperature and outcomes of critically ill patients with CHD. Restricted cubic spline for unadjusted in-hospital mortality (**A**), 28-day mortality (**B**), 90-day mortality (**C**) and adjusted in-hospital mortality (**D**), 28-day mortality (**E**), 90-day mortality (**F**). CI, confidence interval; HR, hazard ratio

To obtain a clearer grasp of how body temperature worked with outcomes, we regarded it as a continuous variable and utilized restricted cubic splines (RCS) to explore whether the duo had linear correlations (Fig. 2). There is a “U-type” relationship between body temperature and outcomes for in-hospital mortality (Fig. 2A), 28-day (Fig. 2B) and 90-day mortality (Fig. 2C). Further adjusting for confounding factors in model 3 (Fig. 2D-2F), the right-hand side of the curve ramped down to the baseline showcasing no statistically increased mortality; while the left hand of that stood a statistically higher mortality though slight as it was. The risk of death went up in the hypothermia group with the decrease of body temperature and elucidated the result of Cox regression analysis a step further.

### Study outcomes

**Fig. 3:**
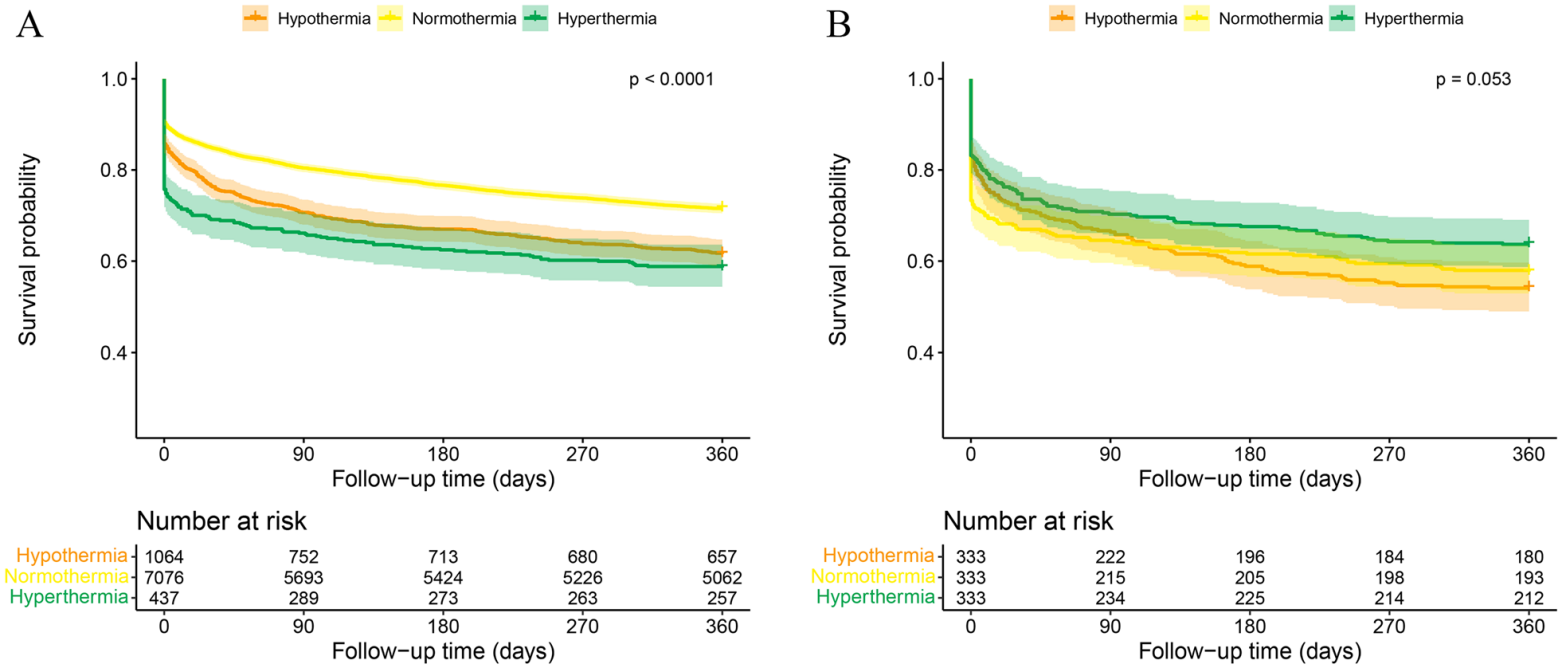
Kaplan–Meier curves of cumulative event-free survival of all-cause death in different groups. Groups were divided by body temperature (℃) in three groups (hypothermia: ≤ 36.5 ℃, normothermia: 36.6–37.4℃, hyperthermia: ≥ 37.5 ℃) unadjusted (**A**) and adjusted by propensity score matching (**B**)

Kaplan–Meier curves (KM curves) was performed before and after propensity score matching (PSA) (Additional File 1: Table S2). The KM curves portrayed a significantly higher risk of death over 1 year in hypothermia and hyperthermia group (log-tank test *P* < 0.0001, Fig. 3A). A dramatic drop occurred in the first 28 days (mostly during the hospital stay) in all groups and turned to a smooth decrease afterward. After PSM, there is no statistically difference among three groups (*P* = 0.053, Fig. 3B).

### Subgroup analysis

Further assessment of the risk stratification value of body temperature as a continuous variable was performed in subgroups consisting of age, gender, ethnicity, heart arrest and cardiogenic shock (Table 3). Our results showed a negative association between body temperature and 28-day or 90-day mortality in subgroups of those aged ≥ 65 years (with adjusted HR ranged from 0.75 to 0.77) and those aged < 65 years (with adjusted HR ranged from 0.66 to 0.72). So they were, for 90-day mortality with those male, white, without heart arrest and with cardiogenic shock as well. Whereas, it was found that the association between body temperature and mortality had been affected by cardiogenic shock (with *P* value for interaction < 0.01).

**Table 3:** Adjusted analysis of association with in-hospital, 28- and 90-day mortality for body temperature

Subgroup		Adjusted HR (95% CI)		*P* value for interaction	Adjusted HR (95% CI)		*P* value for interaction	Adjusted HR (95% CI)		*P* value for interaction
		In-hospital	*P* value		28-day	*P* value		90-day	*P* value	
Age	<65	0.82 (0.58–1.16)	0.27	<0.05	0.72 (0.53–0.97)	0.03	0.02	0.66 (0.51–0.85)	<0.01	0.08
	≥65	0.87 (0.74–1.02)	0.10		0.77 (0.67–0.88)	<0.01		0.75 (0.66–0.85)	<0.01	
Gender	Male	1.03 (0.83–1.27)	0.81	0.08	0.85 (0.71–1.01)	0.07	0.76	0.82 (0.69–0.97)	0.02	0.90
	Female	1.01 (0.76–1.34)	0.97		1.05 (0.82–1.34)	0.72		1.00 (0.79–1.25)	0.97	
Ethnicity	White	1.00 (0.80–1.26)	0.96	0.44	0.85 (0.70–1.03)	0.11	0.20	0.82 (0.69-0.98)	0.03	0.15
	Other	1.04 (0.79–1.37)	<0.01		0.96 (0.5–1.21)	0.71		0.93 (0.75–1.16)	0.55	
Heart arrest	Yes	1.17 (0.78–1.74)	0.45	<0.01	1.25 (0.87–1.78)	0.23	0.25	1.16 (0.83–1.62)	0.39	0.54
	No	0.99 (0.81–1.20)	0.91		0.85 (0.72–1.00)	0.06		0.82 (0.70–0.96)	0.01	
Cardiogenic shock	Yes	0.80 (0.60–1.07)	0.14	<0.01	0.78 (0.60–1.01)	0.06	<0.01	0.74 (0.58–0.95)	0.02	<0.01
	No	1.10 (0.90–1.36)	0.36		0.94 (0.79–1.12)	0.51		0.92 (0.79–1.09)	0.34	

## Discussion

To our knowledge, this is the first study to report the association of low body temperature and worse mortality in critically ill patients with coronary heart disease. In this retrospective cohort study, we analyzed 8577 patients and divided them into three groups: hypothermia (31.8–36.5), normothermia (36.6–37.4), and hyperthermia (37.5–39.6). The methods employed in this study to presage the disease outcomes are based on previous articles [23, 24]. Observing that the hypothermia group had a higher mortality risk over two other groups, we further perceived body temperature as a continuous variable and found a negative relationship between temperatures and worse outcomes. The predictive power of the full model has been examined based on the DCA model, and it was found that body temperature could serve as a convenient outcome predictor for critically ill patients.

Regarding of the intergroup heterogeneity showcased in baseline characteristics table which may cause confounding bias to KM curves, we further adjusted that through PSM. Though post-PSM results correct the probable fallacious outcome in hyperthermia group to some extent (the protective effect of hyperthermia for patients admitted to ICU had been described in former paper [25]), they did not present a statistically higher mortality in hypothermia group.

Intriguingly, in subgroup analysis, we found interactions between body temperature and age, heart arrest or cardiogenic shock. However, only those with cardiogenic shock had both statistically significant simple effect and statistically significant interaction effect in terms of 90-day mortality. Thus, higher proportion of patients with cardiogenic shock history was one of the crucial reasons of why hypothermia group experienced increased mortality especially in long term (the same result illustrated in the adjusted KM curves).

Studies have demonstrated that hypothermia happened due to the changes of thermoregulation system under anesthesia (including abolished behavioral responses, compromised homeostasis and reduced thresholds of vasoconstriction and shivering) [26]. Apart from this, refrigerated liquid drugs used and excessive blood loss in operations and poor physical quality at an older age can lead to hypothermia as well [27]. Systemic hemodynamic depression realized by catecholamine usage in ICU worked together with the thermoregulatory system to cause a lower temperature [28].

It has been investigated that body temperature at admission was related to the outcome and hypothermia appeared to be a significant and independent indicator of increased mortality rates both during ICU stays and over the long term [28]. In addition to that, numerous previous clinical studies have been carried on to research into the association of low body temperature with mortality and morbidity of cardiac patients. According to DeFoe et al. [14], regardless of any sites of core body temperature (nasopharyngeal, esophageal, bladder or rectal), patients undergoing isolated on-pump coronary artery bypass grafting (CABG) surgery have consistently higher in-hospital mortality rates with increasing colder temperatures; moreover, lower temperature groups were found to exhibit greater myocardial injury as assessed by myocardial markers. Likewise, Nam et al. [20] reported that the all-cause mortality of moderate-to-severe hypothermia was more than two times of that in normothermia for off-pump CABG patients and even mild hypothermia (no less than 35.5℃) was found an unsatisfied outcome during the follow-up of 47 months. However, another study that enrolled isolated off-pump CABG patients showed no statistically difference for in-hospital mortality between hypothermia group and normothermia group neither before nor after propensity score matching, while a distinction in postoperative transfusion of red cell concentrates, duration of intubation and ICU stay [15]. Unexpectedly, there was a higher rate of in-hospital mortality in the normothermia group than in the hypothermia group after pairing, though this difference was not statistically significant (*P* = 0.975 vs. *P* = 0.244). Extending to all sorts of cardiac surgeries, the results of multivariable regression analysis showed higher mortality in hypothermia group (body temperature < 36 ℃) during the 1-year follow-up [16]. To figure out the reason for unstable results of in-hospital mortality, Karalapillai et al. [29] differentiated hypothermia into transient type and persistent type in the multi-center observational study, finding that in-hospital mortality was statistically associated with persistent hypothermia but not transient hypothermia. This may shed light on variations of results through different proportions of persistent hypothermia in those studies. Moreover, low body temperature was recognized as an independent marker of poor cardiovascular mortality and rehospitalization in patients admitted with worsening heart failure and reduced ejection fraction [30].

Therapeutic hypothermia triggered by all sorts of cooling strategies, lowering the temperature between 32 ℃ and 35 ℃ for at least 24 h, has been shown to be increasingly used in the post-resuscitation care for post-cardiac arrest patients [31]. Nevertheless, studies showed that no improvement in mortality or neurologic outcome was discovered in the therapeutic hypothermia group over normothermia group [32] and even higher mortality was noticed in patients without cardiac arrest [31]. This reflects distinct causes of low body temperature (no matter for anesthesia-induced or cooling strategy-induced) may have the same tendency on worse outcomes. Similarly, lower ambient temperature, including during cold spells, could elevate mortality and morbidity of cardiovascular disease [33].

Current data about associations between body temperature and patients with coronary heart disease, especially critically ill ones, are limited. In this specific cohort of ICU patients with CHD, we found that low body temperature was an efficient and convenient independent predictor of greater mortality in these patients.

However, this study has several limitations. First, selection bias cannot be evitable due to its retrospective study nature. Since this is a single-center study for confined regions and populations, external validation should be examined by more prospective cohort studies in the future. Second, important indicators such as LVEF and cholesterol levels were not sufficient (less than 15%) in the database. Third, the possible implementation of therapeutic hypothermia in this study may decrease the hazard ratios of mortality for patients in lower temperatures and conceal the probable damage in patients with abnormally high temperature.

## Conclusions

The study showcased critically ill CHD patients with hypothermia after ICU admission had a higher risk of mortality. Measuring body temperature may provide practical evidence for risk stratification and further research is required to testify this.

### Supplementary Information

**Additional file 1: Figure S1.** Distribution of average body temperature within 24 h after ICU admission in coronary heart disease patients. **Table S1.** Missing number and percentage for risk variables. **Table S2.** Post-matched among three body temperature groups.

## Data Availability

The datasets generated and/or analyzed during the current study are available in the MIMIC-IV 2.2 database (https://physionet.org/content/mimiciv/2.2/).
