# Supplementary material for: Low body temperature and mortality in critically ill patients with coronary heart disease: a retrospective analysis from MIMIC-IV database
Source: Eur J Med Res. 2023 Dec 20;28:614. doi: 10.1186/s40001-023-01584-8 (PMC10731844; doi:10.1186/s40001-023-01584-8)
Supplement: Supplementary file 1 — Additional file 1: Figure S1. Distribution of average body temperature within 24 h after ICU admission in coronary heart disease patients. Table S1. Missing number and percentage for risk variables. Table S2. Post-matched among three body temperature groups. [file 40001_2023_1584_MOESM1_ESM.docx]

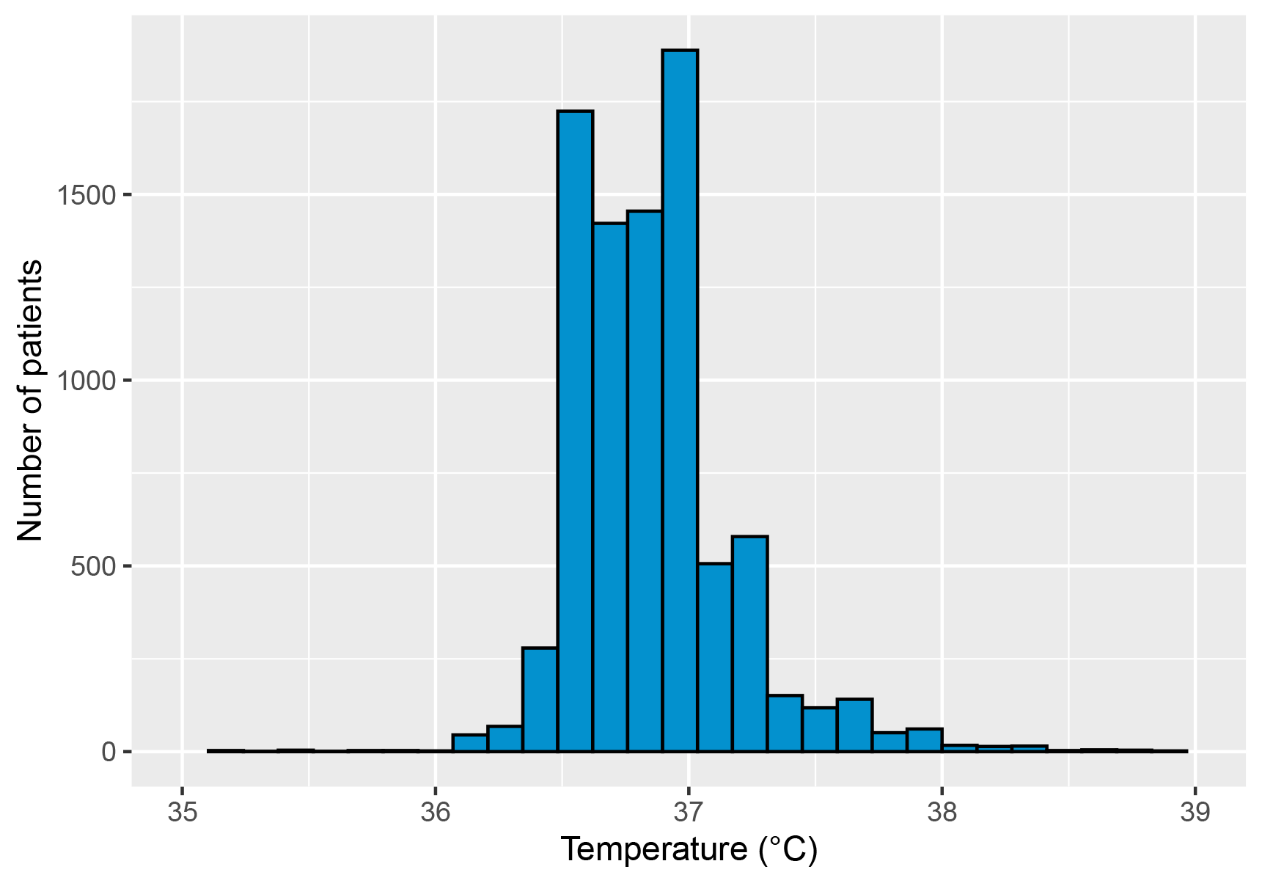


**Supplemental Figure S1** Distribution of average body temperature within 24 hours after ICU admission in coronary heart disease patients.

**Supplemental Table S1 Missing number and percentage for risk variables.**

| Risk variables | Missing number (%) |
| --- | --- |
| Age | 0 (0) |
| Gender | 0 (0) |
| Ethnicity | 3 (0.03) |
| Atrial fibrillation | 0 (0) |
| Chronic kidney disease | 0 (0) |
| COPD or PH | 0 (0) |
| Diabetes mellitus | 0 (0) |
| Hyperlipidemia | 0 (0) |
| Hypertension | 0 (0) |
| Prior myocardial infarction | 0 (0) |
| Previous cardiac surgery | 0 (0) |
| Heart arrest | 0 (0) |
| Cardiogenic shock | 0 (0) |
| WBC | 162 (1.89) |
| Hemoglobin | 163 (1.90) |
| Hematocrit | 160 (1.87) |
| Platelet | 162 (1.89) |
| Serum creatinine | 120 (1.40) |
| Glucose | 161 (1.88) |
| Blood urea nitrogen  INR  Lactate  Potassium  Sodium | 122 (1.42)  874 (10.19)  2562 (29.87)  128 (1.49)  122 (1.42) |
| Admission type | 0 (0) |
| First care unit  SBP  DBP  MBP  Heart rate  Respiratory rate  SOFA  SAPS II  Dobutamine  Dopamine  Epinephrine  Milrinone  Norepinephrine  Vasopressin  Phenylephrine | 0 (0)  6 (0.07)  6 (0.07)  1 (0.01)  0 (0)  6 (0.07)  0 (0)  0 (0)  0 (0)  0 (0)  0 (0)  0 (0)  0 (0)  0 (0)  0 (0) |
| 30-day mortality | 0 (0) |
| 90-day mortality | 0 (0) |
| 180-day mortality | 0 (0) |
| 1-year mortality | 0 (0) |
| Abbreviations: COPD, chronic obstructive pulmonary disease; PH, pulmonary hypertension; WBC, white blood cell; BUN, blood urea nitrogen; INR, international normalized ratio; SBP, systolic blood pressure; DBP, diastolic blood pressure; MBP, mean blood pressure; SOFA, sequential organ failure assessment; SAPS, simplified acute physiology score; SD, standard deviation. | |

**Supplemental Table S2 Post-matched among three body temperature groups.**

| Variables | | Hypothermia (N=333) | Normothermia (N=333) | Hyperthermia (N=333) | *P*-value | Test SMD |
| --- | --- | --- | --- | --- | --- | --- |
| Patient characteristics | |  |  |  |  |  |
|  | Age, years (SD) | 71.74 (13.57) | 64.74 (12.68) | 71.74 (11.39) | <0.001 | 0.372 |
|  | Male, n (%) | 198 (59.5) | 226 (67.9) | 228 (68.5) | 0.024 | 0.126 |
|  | White, n (%) | 218 (65.5) | 198 (59.5) | 246 (73.9) | <0.001 | 0.206 |
| Patient history, n (%) | |  |  |  |  |  |
|  | Atrial fibrillation | 194 (58.3) | 148 (44.4) | 179 (53.8) | 0.001 | 0.186 |
|  | Chronic kidney disease | 168 (50.5) | 118 (35.4) | 161 (48.3) | <0.001 | 0.204 |
|  | COPD or PH | 164 (49.2) | 95 (28.5) | 96 (28.8) | <0.001 | 0.29 |
|  | Diabetes mellitus | 145 (43.5) | 168 (50.5) | 169 (50.8) | 0.109 | 0.096 |
|  | Hyperlipidemia | 100 (30.0) | 79 (23.7) | 72 (21.6) | 0.034 | 0.129 |
|  | Hypertension | 98 (29.4) | 76 (22.8) | 75 (22.5) | 0.066 | 0.105 |
|  | Prior myocardial infarction | 137 (41.1) | 113 (33.9) | 113 (33.9) | 0.083 | 0.1 |
|  | Previous cardiac surgery | 69 (20.7) | 77 (23.1) | 104 (31.2) | 0.005 | 0.161 |
|  | Heart arrest | 43 (12.9) | 39 (11.7) | 25 ( 7.5) | 0.06 | 0.12 |
|  | Cardiogenic shock | 50 (15.0) | 60 (18.0) | 68 (20.4) | 0.189 | 0.095 |
| Laboratory tests | |  |  |  |  |  |
|  | WBC, K/uL (SD) | 14.92 (21.31) | 14.83 (9.68) | 12.56 (5.04) | 0.045 | 0.15 |
|  | Hemoglobin, g/dL (SD) | 9.77 (1.96) | 10.30 (2.07) | 10.03 (1.73) | 0.002 | 0.182 |
|  | Hematocrit, % (SD) | 30.70 (5.78) | 31.96 (6.21) | 30.92 (5.20) | 0.011 | 0.144 |
|  | Platelet, K/uL (SD) | 215.34 (125.27) | 190.30 (106.14) | 168.78 (83.09) | <0.001 | 0.293 |
|  | Serum creatinine, mg/dL (SD) | 1.88 (1.90) | 1.78 (1.54) | 1.77 (1.61) | 0.612 | 0.045 |
|  | Glucose, mg/dL (SD) | 151.19 (59.49) | 167.01 (76.50) | 145.46 (58.57) | <0.001 | 0.215 |
|  | BUN, mg/dL (SD) | 35.84 (25.77) | 29.62 (18.97) | 34.82 (28.67) | 0.003 | 0.175 |
|  | INR (SD) | 1.71 (1.07) | 1.58 (0.79) | 1.59 (0.83) | 0.133 | 0.092 |
|  | Lactate, mg/dL (SD) | 2.15 (1.36) | 2.40 (1.74) | 2.41 (1.77) | 0.069 | 0.109 |
|  | Potassium, mEq/L (SD) | 4.39 (0.55) | 4.31 (0.61) | 4.44 (0.60) | 0.011 | 0.154 |
|  | Sodium, mEq/L (SD) | 138.33 (4.60) | 139.14 (5.57) | 137.03 (4.60) | <0.001 | 0.285 |
| ICU type, n (%) | |  |  |  | <0.001 | 0.863 |
|  | Cardiac Vascular Intensive Care Unit (CVICU) | 62 (18.6) | 51 (15.3) | 165 (49.5) |  |  |
|  | Coronary Care Unit (CCU) | 46 (13.8) | 45 (13.5) | 72 (21.6) |  |  |
|  | Medical Intensive Care Unit (MICU) | 47 (14.1) | 96 (28.8) | 31 ( 9.3) |  |  |
|  | Medical/Surgical Intensive Care Unit (MICU/SICU) | 119 (35.7) | 51 (15.3) | 30 ( 9.0) |  |  |
|  | Neuro Intermediate | 3 ( 0.9) | 0 ( 0.0) | 2 ( 0.6) |  |  |
|  | Neuro Surgical Intensive Care Unit (Neuro SICU) | 6 ( 1.8) | 29 ( 8.7) | 5 ( 1.5) |  |  |
|  | Surgical Intensive Care Unit (SICU) | 21 ( 6.3) | 29 ( 8.7) | 14 ( 4.2) |  |  |
|  | Trauma SICU (TSICU) | 29 ( 8.7) | 32 ( 9.6) | 14 ( 4.2) |  |  |
| Admission type, n (%) | |  |  |  | <0.001 | 0.285 |
|  | Elective | 6 ( 1.8) | 3 ( 0.9) | 15 ( 4.5) |  |  |
|  | Emergency | 125 (37.5) | 142 (42.6) | 89 (26.7) |  |  |
|  | Other | 118 (35.4) | 94 (28.2) | 132 (39.6) |  |  |
|  | Urgent | 84 (25.2) | 94 (28.2) | 97 (29.1) |  |  |
| Vital sign, n (%) | |  |  |  |  |  |
|  | SBP | 117.78 (17.12) | 114.14 (14.61) | 111.31 (12.67) | <0.001 | 0.288 |
|  | DBP | 63.37 (11.33) | 61.20 (10.08) | 59.60 (9.19) | <0.001 | 0.245 |
|  | MBP | 79.05 (11.52) | 76.59 (9.91) | 75.43 (8.36) | <0.001 | 0.238 |
|  | Heart rate | 86.57 (16.44) | 92.21 (16.28) | 79.79 (12.81) | <0.001 | 0.551 |
|  | Respiratory rate | 20.20 (3.42) | 21.47 (3.73) | 18.50 (3.24) | <0.001 | 0.57 |
| Type of CHD, n (%) | |  |  |  | <0.001 | 0.304 |
|  | Acute coronary syndrome | 277 (83.2) | 253 (76.0) | 307 (92.2) |  |  |
|  | Stable coronary heart disease | 56 (16.8) | 80 (24.0) | 26 ( 7.8) |  |  |
| Vasoactive drugs during ICU stay, n (%) | |  |  |  |  |  |
|  | Dobutamine | 18 ( 5.4) | 22 ( 6.6) | 37 (11.1) | 0.014 | 0.139 |
|  | Dopamine | 18 ( 5.4) | 17 ( 5.1) | 23 ( 6.9) | 0.567 | 0.051 |
|  | Epinephrine | 51 (15.3) | 47 (14.1) | 41 (12.3) | 0.53 | 0.058 |
|  | Milrinone | 6 ( 1.8) | 4 ( 1.2) | 13 ( 3.9) | 0.051 | 0.116 |
|  | Norepinephrine | 118 (35.4) | 199 (59.8) | 105 (31.5) | <0.001 | 0.392 |
|  | Vasopressin | 45 (13.5) | 93 (27.9) | 41 (12.3) | <0.001 | 0.265 |
|  | Phenylephrine | 79 (23.7) | 91 (27.3) | 117 (35.1) | 0.004 | 0.168 |
| Score system (SD) | |  |  |  |  |  |
|  | SOFA | 6.59 (3.71) | 8.44 (3.91) | 6.71 (3.54) | <0.001 | 0.328 |
|  | SAPS II | 42.36 (13.20) | 46.53 (15.06) | 42.39 (12.93) | <0.001 | 0.197 |
| Outcome, n (%) | |  |  |  |  |  |
|  | In-hospital mortality | 59 (17.7) | 89 (26.7) | 56 (16.8) | 0.002 | - |
|  | 28-day mortality | 92 (27.6) | 107 (32.1) | 81 (24.3) | 0.079 | - |
|  | 90-day mortality | 112 (33.6) | 118 (35.4) | 99 (29.7) | 0.277 | - |
|  | 1-year mortality | 153 (45.9) | 141 (42.3) | 121 (36.3) | 0.039 | - |
| Abbreviations: COPD, chronic obstructive pulmonary disease; PH, pulmonary hypertension; WBC, white blood cell; BUN, blood urea nitrogen; INR, international normalized ratio; SBP, systolic blood pressure; DBP, diastolic blood pressure; MBP, mean blood pressure; SOFA, sequential organ failure assessment; SAPS, simplified acute physiology score; SD, standard deviation. | | | | | | |
